# Clinical study of Wuwei Fuzheng Yijing formula in the treatment of sperm DNA damage in male infertility: A study protocol for a randomized controlled trial

**DOI:** 10.1097/MD.0000000000031226

**Published:** 2022-10-28

**Authors:** Ninghua Li, Chenming Zhang, Zulong Wang, Qi Zhang, Rubing Chen, Zhong Hua, Shizhong Zhao, Huiyuan Shen, Guifeng Chang, Wenxi Wan

**Affiliations:** a College of the First Clinical Medicine, Henan University of Traditional Chinese Medicine, Zhengzhou, Henan Province, P.R. China; b College of the Second Clinical Medicine, Henan University of Traditional Chinese Medicine, Zhengzhou, Henan Province, P.R. China; c The First Affiliated Hospital of Henan University of Traditional Chinese Medicine, Zhengzhou, Henan Province, P.R. China.

**Keywords:** male infertility, randomized controlled trial, sperm DNA damage, traditional Chinese medicine, Wuwei Fuzheng Yijing formula

## Abstract

The incidence of male infertility is increasing in recent years, and the semen routine examination of some patients is normal, but the semen pathological examination shows that the sperm DNA fragmentation index (DFI) is high, and the patients’ clinical manifestations are infertility or recurrent abortion of their spouses. At present, there is no special treatment for male infertility caused by high DFI, and traditional Chinese medicine compound prescription shows potential value in the treatment of male infertility. Wuwei Fuzheng Yijing formula (WFY) is an effective prescription for the treatment of sperm DNA damage in male infertility, but there is no strict clinical trial to support its application. Therefore, we designed a randomized controlled trial to evaluate the efficacy and safety of WFY in patients with sperm DNA damage in male infertility.

**Methods:**

In this randomized controlled study, 100 patients who met the inclusion criteria were randomly divided into WFY group and levocarnitine oral solution group. The treatment period was 12 weeks. The main observation index was sperm DFI, and the secondary observation index was sperm concentration, motility, survival rate, and TCM syndrome score. Safety observation indicators will include electrocardiogram, blood tests (including blood routine tests, liver and renal function), routine urine tests, and routine stool tests. All results were evaluated at the 4th and 8th week of the baseline, and the end of treatment.

**Discussion:**

This study will provide a basis for the efficacy and safety of WFY in the treatment of sperm DNA damage in male infertility with spleen and kidney qi deficiency and blood stasis.

## 1. Introduction

Couples of childbearing age, cohabiting for more than 1 year after marriage, have a normal sexual life, do not use or accept any contraceptive measures, and the woman is normal. Due to the male factor, the woman is fertile, which is called male infertility.^[1]^ In recent years, with the change of people’s lifestyle and the aggravation of environmental pollution, there are more and more male infertility patients.^[2]^ Studies have shown that,^[3,4]^ the incidence of infertility in couples of childbearing age is about 8% to 15%, of which about 50% is caused by male factors. However, more than 30% of patients with male infertility have normal semen routine examination, and semen examination is easily affected by many factors such as ambient temperature, retention time, and subjectivity of testers.^[5]^ Therefore, there are some limitations in evaluating male fertility only based on sperm motility, sperm density, sperm normal shape rate, sperm survival rate, and so on in semen analysis.^[6,7]^

Sperm DNA is the carrier of genetic material. Sperm chromosome abnormality or DNA damage caused by any factor will reduce male fertility potential and even cause male infertility. Sperm DNA fragmentation index (DFI), as an index to evaluate male sperm quality at molecular level, can reflect the degree of sperm DNA damage to some extent, and is of great significance for the objective and scientific evaluation of male fertility.^[8]^ At present, it is generally believed that DFI ≤ 15% is normal, 15% ＜ DFI < 30% is general, if DFI ≥ 30%, sperm DNA integrity is poor, high DFI may lead to biochemical pregnancy, threatened abortion, and recurrent abortion, affecting the pregnancy outcome.^[9–11]^

At present, the main causes of sperm DNA damage are varicocele, elevated testicular temperature, radiotherapy and chemotherapy, environmental factors, and improper lifestyle.^[12]^ Because the etiology of sperm DNA damage is complex and the pathogenesis is unknown, there is no specific treatment at present. Antioxidants such as L-carnitine, vitamin E, and lipoic acid are often used to treat sperm DNA damage in male infertility.^[13]^ However, the therapeutic potential of antioxidants is still controversial due to the limitation of sample size. In addition, these synthetic antioxidants have the disadvantages of high molecular weight, difficult to absorb and expensive. About half of the patients hope to seek more alternative treatments, such as traditional Chinese medicine (TCM) to treat sperm DNA damage in male infertility.

In recent years, clinical studies have found that traditional Chinese medicine plays an important role in the treatment of sperm DNA damage in male infertility, and can improve sperm motility and quantity.^[14]^ Wuwei Fuzheng Yijing formula (WFY) is made from Wuzi Yanzong Pill, a classic prescription for the treatment of male infertility, which is composed of *Radix Astragali*, *Fructus Lycii*, *Achyranthes bidentata*, *Schisandra chinensis*, and *Plantago asiatica*. It has the effect of tonifying kidney, invigorating spleen and tonifying qi, activating blood circulation and removing blood stasis. Modern pharmacological studies show that astragalus polysaccharides and astragalus extract can reduce the level of oxidative stress (OS) and improve energy metabolism.^[15]^ *Lycium barbarum* polysaccharides, *Lycium barbarum* polyphenols and trace elements can inhibit spermatogenic cell apoptosis, enhance cellular immunity, regulate the balance of sex hormones, and so on.^[16]^ Schisandrin B in Schisandra chinensis can increase the level of SOD, reduce the release of lactate dehydrogenase, MDA and reactive oxygen species, and directly scavenge free radicals and play an antioxidant role.^[17]^ The polysaccharide from plantain seed has the effect of antioxidation and scavenging oxygen free radicals.^[18]^ The extract of Achyranthes bidentata can significantly enhance the activity of antioxidant enzymes and the ability of scavenging superoxide anion free radicals, and inhibit the formation of lipid peroxidation of malondialdehyde.^[19]^ The previous animal experiments of the research group showed that Wuwei Fuzheng Yijing Decoction can effectively reduce the DFI of mouse sperm and improve the reproductive capacity of mice.^[20]^

However, the current clinical studies have limitations in methods and sample size, and lack sufficient evidence for WFY as a supplementary treatment for sperm DNA damage in male infertility. Therefore, we aim to design a randomized controlled trial to evaluate the efficacy and safety of WFY in the treatment of sperm DNA damage in male infertility.

## 2. Hypothesis

The hypothesis is that intervention with WFY is better than levocarnitine oral solution (LOS) in these outcomes of sperm DNA damage. We also expect to prove that WFY can be an ideal safe and conservative alternative for patients with sperm DNA damage.

## 3. Methods

This study protocol was developed under the Recommendations for Interventional Trials 2013 Statement (SPIRIT 2013) ^[11]^ (Table 1).

**Table 1:** SPIRIT 2013 Checklist: recommended items to address in a clinical trial protocol and related documents*

Section/item	Item No	Description	Addressed on page number
**Administrative information**			
Title	1	Descriptive title identifying the study design, population, interventions, and, if applicable, trial acronym	1
Trial registration	2a	Trial identifier and registry name. If not yet registered, name of intended registry	2
	2b	All items from the World Health Organization Trial Registration Data Set	N/A
Protocol version	3	Date and version identifier	N/A
Funding	4	Sources and types of financial, material, and other support	11
Roles and responsibilities	5a	Names, affiliations, and roles of protocol contributors	11
	5b	Name and contact information for the trial sponsor	N/A
	5c	Role of study sponsor and funders, if any, in study design; collection, management, analysis, and interpretation of data; writing of the report; and the decision to submit the report for publication, including whether they will have ultimate authority over any of these activities	N/A
	5d	Composition, roles, and responsibilities of the coordinating center, steering committee, endpoint adjudication committee, data management team, and other individuals or groups overseeing the trial, if applicable (see Item 21a for data monitoring committee)	N/A
**Introduction**			
	6a	Description of research question and justification for undertaking the trial, including summary of relevant studies (published and unpublished) examining benefits and harms for each intervention	2-3
Background and rationale	6b	Explanation for choice of comparators	3
Objectives	7	Specific objectives or hypotheses	3
Trial design	8	Description of trial design including type of trial (e.g., parallel group, crossover, factorial, single group), allocation ratio, and framework (e.g., superiority, equivalence, noninferiority, exploratory)	4
**Methods: Participants, interventions, and outcomes**			
Study setting	9	Description of study settings (e.g., community clinic, academic hospital) and list of countries where data will be collected. Reference to where list of study sites can be obtained	4-5
Eligibility criteria	10	Inclusion and exclusion criteria for participants. If applicable, eligibility criteria for study centers and individuals who will perform the interventions (e.g., surgeons, psychotherapists)	5-6
Interventions	11a	Interventions for each group with sufficient detail to allow replication, including how and when they will be administered	6-7
	11b	Criteria for discontinuing or modifying allocated interventions for a given trial participant (e.g., drug dose change in response to harms, participant request, or improving/worsening disease)	7
	11c	Strategies to improve adherence to intervention protocols, and any procedures for monitoring adherence (e.g., drug tablet return, laboratory tests)	7-8
	11d	Relevant concomitant care and interventions that are permitted or prohibited during the trial	6
Outcomes	12	Primary, secondary, and other outcomes, including the specific measurement variable (e.g., systolic blood pressure), analysis metric (e.g., change from baseline, final value, time to event), method of aggregation (e.g., median, proportion), and time point for each outcome. Explanation of the clinical relevance of chosen efficacy and harm outcomes is strongly recommended	7
Participant timeline	13	Time schedule of enrollment, interventions (including any run-ins and washouts), assessments, and visits for participants. A schematic diagram is highly recommended (see Fig. 1)	Figure 1
	14	Estimated number of participants needed to achieve study objectives and how it was determined, including	4
		clinical and statistical assumptions supporting any sample size calculations	
	15	Strategies for achieving adequate participant enrollment to reach target sample size	4-5
	16a	Method of generating the allocation sequence (e.g., computer-generated random numbers), and list of any factors for stratification. To reduce predictability of a random sequence, details of any planned restriction (e.g., blocking) should be provided in a separate document that is unavailable to those who enroll participants or assign interventions	6
	16b	Mechanism of implementing the allocation sequence (e.g., central telephone; sequentially numbered, opaque, sealed envelopes), describing any steps to conceal the sequence until interventions are assigned	6
	16c	Who will generate the allocation sequence, who will enroll participants, and who will assign participants to interventions	6
	17a	Who will be blinded after assignment to interventions (e.g., trial participants, care providers, outcome assessors, data analysts), and how	6
	17b	If blinded, circumstances under which unblinding is permissible, and procedure for revealing a participant’s allocated intervention during the trial	6
	18a	Plans for assessment and collection of outcome, baseline, and other trial data, including any related processes to promote data quality (e.g., duplicate measurements, training of assessors) and a description of study instruments (e.g., questionnaires, laboratory tests) along with their reliability and validity, if known.	7
		Reference to where data collection forms can be found, if not in the protocol	
	18b	Plans to promote participant retention and complete follow-up, including list of any outcome data to be collected for participants who discontinue or deviate from intervention protocols	7
	19	Plans for data entry, coding, security, and storage, including any related processes to promote data quality	7-8
		(e.g., double data entry; range checks for data values). Reference to where details of data management procedures can be found, if not in the protocol	
	20a	Statistical methods for analyzing primary and secondary outcomes. Reference to where other details of the statistical analysis plan can be found, if not in the protocol	8
	20b	Methods for any additional analyses (e.g., subgroup and adjusted analyses)	8
	20c	Definition of analysis population relating to protocol non-adherence (e.g., as randomized analysis), and any statistical methods to handle missing data (e.g., multiple imputation)	8
	21a	Composition of data monitoring committee (DMC); summary of its role and reporting structure; statement of whether it is independent from the sponsor and competing interests; and reference to where further details about its charter can be found, if not in the protocol. Alternatively, an explanation of why a DMC is not needed	N/A
	21b	Description of any interim analyses and stopping guidelines, including who will have access to these interim results and make the final decision to terminate the trial	N/A
	22	Plans for collecting, assessing, reporting, and managing solicited and spontaneously reported adverse events and other unintended effects of trial interventions or trial conduct	N/A
	23	Frequency and procedures for auditing trial conduct, if any, and whether the process will be independent from investigators and the sponsor	N/A
	24	Plans for seeking research ethics committee/institutional review board (REC/IRB) approval	10-11
	25	Plans for communicating important protocol modifications (e.g., changes to eligibility criteria, outcomes, analyses) to relevant parties (e.g., investigators, REC/IRBs, trial participants, trial registries, journals, regulators)	N/A
	26a	Who will obtain informed consent or assent from potential trial participants or authorized surrogates, and how (see Item 32)	7-8
	26b	Additional consent provisions for collection and use of participant data and biological specimens in ancillary studies, if applicable	N/A
	27	How personal information about potential and enrolled participants will be collected, shared, and maintained in order to protect confidentiality before, during, and after the trial	7-8
	28	Financial and other competing interests for principal investigators for the overall trial and each study site	11
	29	Statement of who will have access to the final trial dataset, and disclosure of contractual agreements that limit such access for investigators	11
	30	Provisions, if any, for ancillary and post-trial care, and for compensation to those who suffer harm from trial participation	N/A
	31a	Plans for investigators and sponsor to communicate trial results to participants, healthcare professionals, the public, and other relevant groups (e.g., via publication, reporting in results databases, or other data sharing arrangements), including any publication restrictions	11
	31b	Authorship eligibility guidelines and any intended use of professional writers	N/A
	31c	Plans, if any, for granting public access to the full protocol, participant-level dataset, and statistical code	N/A
	32	Model consent form and other related documentation given to participants and authorized surrogates	N/A
	33	Plans for collection, laboratory evaluation, and storage of biological specimens for genetic or molecular analysis in the current trial and for future use in ancillary studies, if applicable	N/A

* It is strongly recommended that this checklist be read in conjunction with the SPIRIT 2013 Explanation & Elaboration for important clarification on the items. Amendments to the protocol should be tracked and dated. The SPIRIT checklist is copyrighted by the SPIRIT Group under the Creative Commons “Attribution-NonCommercial-NoDerivs 3.0 Unported” license.

### 3.1. Study design and setting

**Figure 1.**
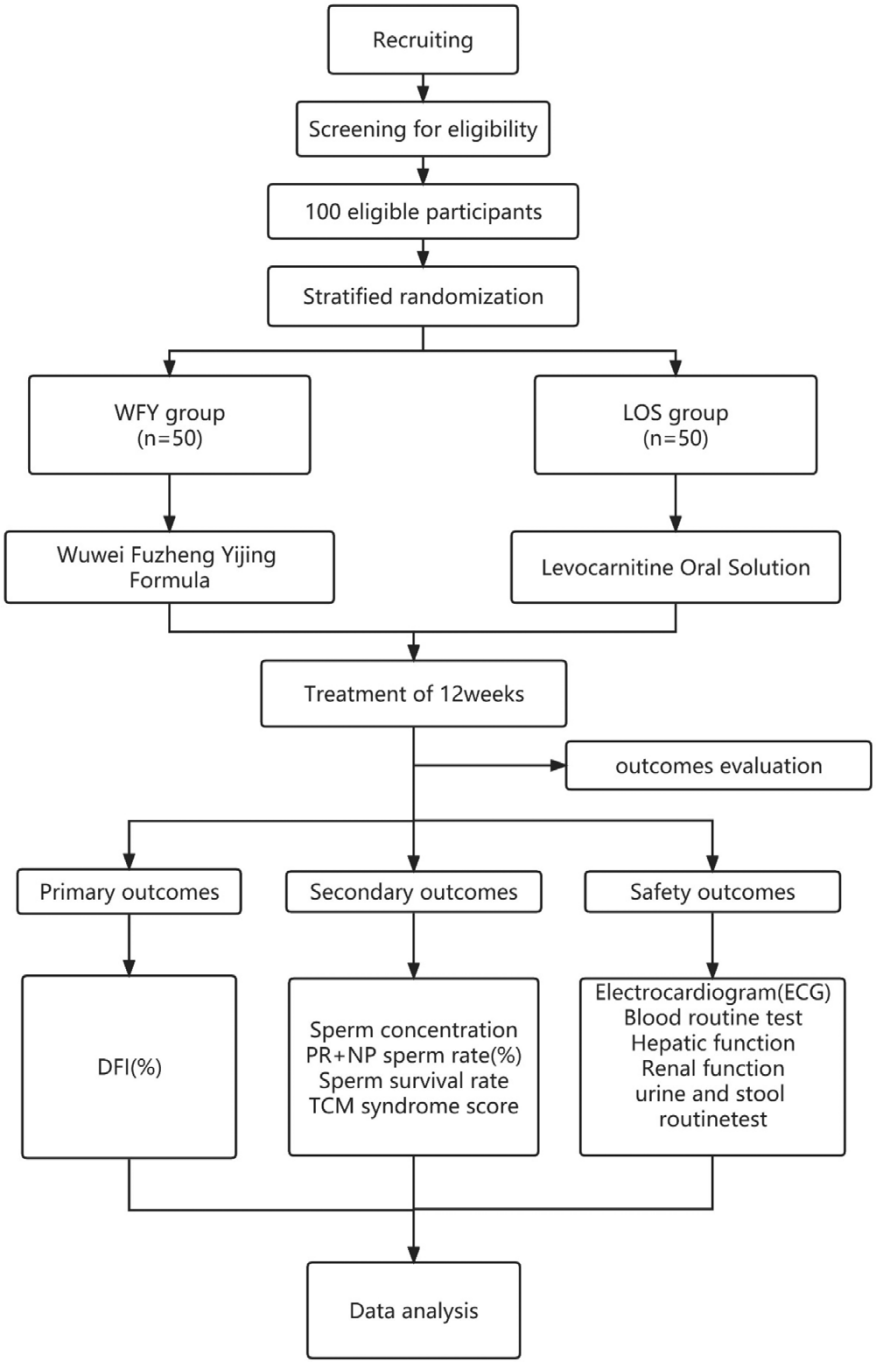
Flow chart of the study design.

This study is a 12-week single-center, evaluator-blind, randomized controlled trial. To evaluate the efficacy and safety of WFY and LOS in the treatment of sperm DNA damage in male infertility. The experiment will be conducted in the male Department of the first affiliated Hospital of Henan University of traditional Chinese Medicine. All participants will be required to provide a written informed consent form before entering the trial. Details of the research plan are shown in Table 1. The flow chart of the test is shown in Figure 1.

### 3.2. Sample size

Sample size was estimated based on the primary study results. Our previous pre-experiment have shown that the effective rate (*P*_1_) of the WFY group (treatment group) is 81%, and the effective rate (*P*_2_) of the LOS group (control group) is 50%. The following formula was used to estimate sample size: $n=\frac{{\left(Z_{1-\alpha /2}+Z_{1-\beta }\right)}^{2}[P_{1}(1-P_{1})+P_{2}(1-P_{2})]}{{\left(P_{1}-P_{2}\right)}^{2}}$ with a 5% significance level (α= 0.05, two-sided) and 90% power (β= 0.1), the final sample size has been set at a total of 100 patients (50 in each group), assuming a 10% dropout rate.

### 3.3. Recruitment and participants

The recruitment of patients started on June 1, 2022, and it is expected that by May 31, 2023, the required sample size will be reached. This protocol was submitted before completion of recruitment. A total of 100 patients with sperm DNA damage in male infertility will be included in this study after obtaining informed consent. We will post recruitment posters in hospitals and recruitment messages on hospital social media to recruit participants. All participants should meet the diagnostic criteria of sperm DNA damage in male infertility and TCM syndrome diagnosis criteria of spleen and kidney qi deficiency and blood stasis type. Patients who meet the study criteria will conduct the study in the Department of Men’s Department of the first affiliated Hospital of Henan University of traditional Chinese Medicine. Eligible participants will be randomly assigned to the WFY group or LOS group at 1:1. The treatment will last for 12 weeks, and the relevant indicators will be evaluated at the end of treatment.

### 3.4. Diagnostic criteria

The diagnostic criteria of sperm DNA damage in male infertility will be based on the 6th edition of the World Health Organization Human semen testing and processing Laboratory Manual (2), which should meet the following two criteria: (a) couples of childbearing age, cohabitation after marriage for more than 1 year, normal sexual life, did not use or accept any contraceptive measures, the woman is normal, due to male factors caused by female infertility, known as male infertility. (b) DFI ≥ 30%.

The TCM pattern differentiation criteria of spleen and kidney qi deficiency and blood stasis syndrome will refer to the Guiding Principles of New Herbs Research,^[21]^ and the Guidelines for Diagnosis and Treatment of Male Infertility with Integrated Traditional Chinese and Western Medicine (Trial Edition).^[22]^ The standards of spleen and kidney qi deficiency and blood stasis syndrome were separated into major symptoms and secondary symptoms. Major symptoms: Couples of childbearing age who have lived together for more than one year, have regular sexual life, not taken any contraceptive measures, and the woman cannot become pregnant due to the man’s reasons. Secondary symptoms: (a) soreness and weakness of waist and knees, Physical fatigue and fatigue; (b) dizziness and tinnitus, lack of diet and poor digestive function; (c) dark tongue body with ecchymosis or petechia, the tongue coating is thin and white; (d) thick and tortuous sublingual veins; and (e) a deep and weak pulse, or a deep and unsmooth pulse. Patients were considered to have spleen and kidney qi deficiency and blood stasis syndrome if they met all of the major symptoms and two secondary symptoms such as a and c, or a and d, or b and c, or b and d, and met the pulse manifestation.

### 3.5. Inclusion criteria

(a) Male, aged from 22 to 45 years old; (b) diagnosed as sperm DNA damage of male infertility; (c) accorded with the syndrome differentiation standard of spleen and kidney qi deficiency and blood stasis syndrome; (d) normal sexual life and regular sexual life (the frequency of sexual life was required not less than once a week); (e) willing to join this study and sign the informed consent form.

### 3.6. Exclusion criteria

(a) Infertility caused by organic lesions of the reproductive system or female infertility; (b) infertility caused by the inability to complete sexual intercourse, including, but not limited to, erectile dysfunction and ejaculation disorders; (c) genitourinary tract obstruction and infection, such as chlamydia trachomatis or mycoplasma infection; (d) chromosome abnormalities (including karyotype abnormalities and Y chromosome microdeletions); (e) patients with a history of drug allergy; (f) with abnormal sex hormone and seminal plasma biochemical examination; (g) with primary diseases such as severe cardiovascular, liver, kidney, and hematopoietic system, and unable to cooperate with treatment; (h) using drugs affecting experimental research within 3 months; (i) participated in other clinical trials in the past 3 months.

### 3.7. Randomization and blinding

The patients who met the inclusion criteria and signed the informed consent form were randomly divided into WFY group and LOS group according to 1:1. The WFY group would be treated with WFY and the LOS group would be treated with LOS for 12 weeks. Randomization is based on a list of random numbers generated by the SPSS26.0 software (IBM Corp, Armonk, NY). An independent researcher puts the random number table in an opaque envelope that contains the allocation number and is responsible for hiding the allocation order. Because of the particularity of the intervention, it is impossible to blind the participants in the trial, and the results evaluators, data managers and statisticians do not know the distribution of treatment.

### 3.8. Interventions

#### 3.8.1. Basic interventions.

The 2 groups were given basic interventions, including: (a) telling patients to give up smoking and alcohol during medication, forbidding sauna and basin (pool) bath, avoiding exposure to high temperature, radiation pollution, chemical poison pollution, and other environments; (b) regular work and rest during treatment to relax.

#### 3.8.2. Drug intervention.

##### 3.8.2.1. WFY group.

WFY is composed of Astragalus membranous 30 g, Chinese wolfberry 15 g, Achyranthes bidentata 15 g, Schisandra chinensis 15 g, and plantain seed 12 g. Participants were asked to take 1 g twice a day for 0.5 h after breakfast and dinner for 12 weeks. Traditional Chinese medicine is produced by Sichuan New Green Pharmaceutical Science and Technology Development Co., Ltd., and the production process meets the Good Manufacturing Practice standard.

##### 3.8.2.2. LOS group.

Participants in this group will be required to take the LOS, 10 mL/piece, twice a day, orally during meals. LOS is produced by Shenyang first Pharmaceutical Co., Ltd., Northeast Pharmaceutical Group. 12 weeks is one course of treatment and the participants will receive one course of medication. All drugs will be used should be the same lot number.

##### 3.8.2.3. Combined treatment regulations

If the participants meet the inclusion criteria, and they with other diseases need to continue to use drugs in clinical trials, or if they really need to use other drugs or treatments because of the need for treatment, the name (or treatment), dosage, times, and time of the drugs used should be recorded in detail in the case report form. (a) during the trial, other drugs that affect the evaluation of drug efficacy shall not be used, such as other proprietary Chinese medicine and western medicine for the treatment of male infertility, including various external treatments, etc. (b) Antibiotics shall not be used during the trial. (c) The drugs and treatments that must be taken for other diseases must be recorded in detail in the combined medication list. (d) If the condition gets worse after taking the drug, please use other treatment methods according to the condition of the patient, and the case will be treated ineffectively.

### 3.9. Primary outcome

The main result of this trial was the sperm DFI before and after the treatment.

### 3.10. Secondary outcomes

The secondary results of this experiment include sperm concentration, motility, survival rate, and TCM syndrome score.

### 3.11. Safety outcomes

Safety observation indicators will include electrocardiogram, blood tests (including blood routine tests, liver and renal function), routine urine tests, and routine stool tests. WFY is a compound prescription of traditional Chinese medicine which has been used in clinic for many years. If an adverse event occurs, the clinical researcher will record it in detail in the case report form (including symptoms, time of occurrence, duration, examination, and results). Serious adverse reactions were reported to the Ethics Committee of the first affiliated Hospital of Henan University of traditional Chinese Medicine, and the rescue procedure was started immediately.

### 3.12. Quality control and trial monitoring

#### 3.12.1. Quality control of clinical trials.

(a) The researchers participating in clinical trials must have the professional expertise, qualifications and abilities of clinical trials, and the personnel requirements are relatively fixed after qualification examination. (b) The personnel participating in the clinical trial should be trained uniformly so that the researchers can have a full understanding of the clinical trial scheme and the specific connotation of each index. (c) The researchers should record the contents of the case report form truthfully, in detail and carefully according to the requirements of the case report form, so as to ensure that the contents of the case report form are true and reliable. (d) All observations and findings in clinical trials should be verified to ensure the reliability of the data. (e) Laboratory quality control measures: hospital laboratories establish unified experimental testing index standards, operating procedures, and quality control procedures. Special inspection items must be taken care of by special personnel. (f) Drug counting method was used to monitor the compliance of the subjects. During each follow-up, patients are required to bring the remaining experimental drugs, and the doctor counts the number of drugs personally, records and calculates compliance on the case. Drug compliance = [(dosage-residual)/ prescription] × 100%.

#### 3.12.2. Quality assurance of clinical trials.

(a) The sponsor shall appoint an inspector, and regularly visit the research institution during the clinical trial to ensure that the rights and interests of the subjects in the clinical trial are protected, and that the data recorded and reported are accurate and complete, ensure that the trial follows the approved scheme, drug clinical trial management norms, and relevant laws and regulations. (b) The drug supervision and administration department and the sponsor may entrust inspectors to systematically inspect the activities and documents related to the clinical trial, so as to evaluate whether the test is carried out in accordance with the test plan, standard operating procedures and relevant laws and regulations, whether the test data are recorded timely, truly, accurately and completely. (c) The relevant materials and documents (including medical records) of medical institutions and laboratories participating in clinical trials shall be inspected by the drug supervision and administration department.

### 3.13. Statistical analysis

Professionals will use Statistical Product and Service Solutions (SPSS26.0, International Business Machines Corp., Armonk, NY) software to analyze all data. The sample rate (total effective rate) was compared by χ^2^ test. The grade data (clinical efficacy) were compared by Z test. The metrological data (TCM syndrome score, sperm DFI, main parameters of semen analysis, etc.) were expressed as mean ± standard deviation （χ±S）. If it conformed to normality and homogeneity of variance, independent sample t-test was used for inter-group comparison, paired sample *t*-test was used for intra-group comparison, and rank sum test was used for non-conformity. Correlation analysis uses Pearson correlation analysis. All the statistical tests will be conducted by bilateral difference tests, and if a *P* < .05, the data difference will be considered statistically significant.

## 4. Discussion

In recent years, the incidence of male infertility has increased worldwide, and sperm DNA damage is an important cause of male infertility.^[23,24]^ The genomic sequence information carrying genetic material is located on DNA. During fertilization, the sperm transmits the genetic material in the nucleus to the oocyte and fuses it with the genetic material of the oocyte to form a diploid fertilized egg, which develops into an embryo and continues to the next generation.^[25]^ Therefore, protecting the integrity of sperm DNA is very important for the continuation of life. However, the etiology of male infertility caused by sperm DNA damage is complex and the pathogenesis is not clear, so it is a complicated treatment for male infertility. At present, the treatment of sperm DNA damage is mainly the use of antioxidants, the supplement of antioxidants is beneficial to deal with the damage caused by OS.^[26]^ However, many drugs used to treat sperm DNA damage are empirically managed, and the data on drug efficacy are highly heterogeneous. There is no specific treatment to reduce sperm DNA damage in modern medicine. Therefore, it is of great significance to seek complementary alternative therapy such as Chinese herbal medicine for the treatment and fertility protection of infertile men.

Traditional Chinese medicine (TCM) has been used in the clinical practice of male infertility for hundreds of years.^[27]^ A number of studies have shown that the pathogenesis of male infertility is mainly related to the dysfunction of kidney, liver, and spleen, involving pathological products such as phlegm, water dampness, and blood stasis.^[28–30]^ By tonifying kidney and tonifying qi, invigorating spleen and removing dampness, activating blood circulation and removing blood stasis, and other ways to coordinate viscera function and balance body yin and yang, so as to play a role in the treatment of diseases.

Syndrome differentiation and treatment is the most representative feature of traditional Chinese medicine in the diagnosis and treatment of diseases. Therefore, we take TCM syndrome as one of the inclusion criteria of participants, that is, spleen and kidney qi deficiency and blood stasis syndrome. TCM often uses herbs for tonifying spleen and kidney in the treatment of male infertility, these herbs have the effects of nourishing yin and strengthening yang, tonifying kidney and essence, and can regulate reproductive hormones and promote sperm growth, movement, and maturation. In addition, herbs for promoting blood circulation and removing blood stasis are also widely used, which have the effect of promoting blood circulation, restoring microvascular circulation, improving tissue ischemia, and hypoxia, so as to improve spermatogenic environment, increase sperm motility and promote sperm production.

WFY is an effective compound prescription of traditional Chinese medicine for the treatment of sperm DNA damage in male infertility with spleen and kidney qi deficiency and blood stasis syndrome. Modern pharmacological research shows that traditional Chinese medicine such as Radix Astragali, Chinese wolfberry and Schisandra chinensis can protect male spermatogenic function and improve their fertility; traditional Chinese medicine such as Achyranthes bidentata can effectively improve the blood circulation of related organs of reproductive system, alleviate OS damage caused by excessive reactive oxygen species accumulation, reduce sperm DNA damage and improve pregnancy outcome. The active components of WFY Chinese herbal medicine can play the role of antioxidant stress, scavenging free radicals, inhibiting spermatogenic cell apoptosis, enhancing cellular immunity, and regulating sex hormone balance.

At present, there is a lack of high-quality traditional Chinese medicine research and evidence on sperm DNA damage in male infertility. Therefore, we are conducting a randomized controlled trial to evaluate the efficacy and safety of WFY in the treatment of sperm DNA damage in male infertility. This study is a parallel randomized controlled trial of positive drugs, and we chose LOS as the positive drug, because current studies have shown that LOS can improve DFI and semen analysis parameters in patients with male infertility without serious adverse reactions.^[31]^ We use DFI as the main therapeutic index, sperm concentration, motility, survival rate, and TCM syndrome score as secondary indicators to provide more reference for the evaluation of therapeutic effect. Safety observation indicators will include electrocardiogram, blood tests (including blood routine tests, liver, and renal function), routine urine tests, and routine stool tests. All results will be evaluated at the 4th and 8th week of the baseline and after the end of treatment, providing a reliable basis for efficacy evaluation and adverse reaction observation.

Because of the nature of the intervention, this experiment cannot completely cause blindness. We will ensure that outcome evaluators, data collectors, and analysts are not aware of treatment allocation and strictly follow inclusion and exclusion criteria to improve the homogeneity of subjects. We hope the results will provide preliminary evidence for the efficacy and safety of WFY in the treatment of sperm DNA damage in male infertility with spleen and kidney qi deficiency and blood stasis syndrome.

## 5. Limitation

First, the extrapolation of the research conclusions of RCT to clinical practice is faced with challenges, for example, limited sample size. Besides, it is difficult to implement traditional RCT during patient follow-up.

## Acknowledgments

We would like to thank all the patients who will participate in the trial and the staff for their support.

## Author contributions

**Conceptualization:** Ninghua Li, Chenming Zhang, Zulong Wang.

**Investigation:** Zhong Hua, Rubing Chen, Shizhong Zhao, Huiyuan Shen, Guifeng Chang, Wenxi Wan.

**Supervision:** Chenming Zhang, Rubing Chen, Qi Zhang.

**Writing – original draft:** Ninghua Li, Chenming Zhang.

**Writing – review and editing:** Ninghua Li, Zulong Wang.
